# Combining Max pooling-Laplacian theory and *k-*means clustering for novel camouflage pattern design

**DOI:** 10.3389/fnbot.2022.1041101

**Published:** 2022-11-18

**Authors:** Minhao Wan, Dehui Zhao, Baogui Zhao

**Affiliations:** Department of Airfield and Construction Engineering, Aviation Engineering School, Air Force Engineering University, Xi'an, China

**Keywords:** camouflage, camouflage design, *k-*means clustering, Max pooling, computer aided design

## Abstract

Camouflage is the main means of anti-optical reconnaissance, and camouflage pattern design is an extremely important step in camouflage. Many scholars have proposed many methods for generating camouflage patterns. *k*-means algorithm can solve the problem of generating camouflage patterns quickly and accurately, but *k*-means algorithm is prone to inaccurate convergence results when dealing with large data images leading to poor camouflage effects of the generated camouflage patterns. In this paper, we improve the *k*-means clustering algorithm based on the maximum pooling theory and Laplace's algorithm, and design a new camouflage pattern generation method independently. First, applying the maximum pooling theory combined with discrete Laplace differential operator, the maximum pooling-Laplace algorithm is proposed to compress and enhance the target background to improve the accuracy and speed of camouflage pattern generation; combined with the *k*-means clustering principle, the background pixel primitives are processed to iteratively calculate the sample data to obtain the camouflage pattern mixed with the background. Using color similarity and shape similarity for evaluation, the results show that the combination of maximum pooling theory with Laplace algorithm and *k*-means algorithm can effectively solve the problem of inaccurate results of *k*-means algorithm in processing large data images. The new camouflage pattern generation method realizes the design of camouflage patterns for different backgrounds and achieves good results. In order to verify the practical application value of the design method, this paper produced test pieces based on the designed camouflage pattern generation method and tested the camouflage effect of camouflage pattern in sunny and cloudy days respectively, and the final test results were good.

## Introduction

Modern reconnaissance threats to ground-based military facilities are mainly derived from air-based unmanned reconnaissance and space-based satellite reconnaissance. Due to the longer detection range and high load requirements, this provides the possibility for ground targets to carry out deformation camouflage. The core problem of camouflage is to solve the integration problem of camouflage patterns and the surrounding background environment. How to quickly and accurately generate camouflage patterns has become a hot and difficult point of current research.

Many scholars have conducted many studies on camouflage design and improvement. Wei et al. (2021) generated camouflage patterns using a combination of convolutional kernels and clustering algorithms. Du et al. (2012) designed a method to generate camouflage patterns through parameter control. Xue et al. (2016) designed digital camouflage by the recursive overlap of pattern templates Yang and Yin (2014) developed a target camouflage pattern generation method by using a *k-*means algorithm to extract the background primary color and combine it with a color similarity control algorithm. Yunxiang et al. (2019) proposed a digital camouflage pattern design method for a three-dimensional model of equipment, which is a good guide for the camouflage of ground equipment. Yong et al. (2009) used an improved *k-*means algorithm for camouflage color selection and patching, based on which bionic camouflage design is carried out. Xiao et al. (2021) propose a camouflage generation algorithm based on rectangular block scrambling and a fuzzy C-mean (FCM) clustering method.

A comprehensive study of camouflage pattern design by different scholars shows that the *k-*means clustering method is widely used in camouflage pattern design because of the advantage of simple and fast background pattern primary color extraction, but the *k-*means algorithm is difficult to obtain the global optimal solution due to the inaccuracy of iterative results when processing large data images and is affected by noise points (Yu and Shuang, 2012). There is a need to provide a fast and efficient image compression enhancement method combined with *k-*means to improve the accuracy of the output results of the camouflage pattern design method.

In this paper, we propose a new artifactual image design method for the problem that the *k-*means clustering algorithm is inaccurate in processing the iterative results of big data images and leads to the poor artifactual effect of the generated images. Chapter 2 of this paper presents the Max pooling-Laplacian algorithm. The Max pooling-Laplacian algorithm is used to pre-process the initial background data, Max pooling mainly focuses on compressing the background image to reduce the amount of data and remove undesirable noise without changing the key features of the image, and the Laplacian algorithm enhances the image features to make the background image simple and easy to handle. Chapter 3 of this paper introduces the *k-*means and *k-*means++ algorithms. The clustering algorithm is chosen to use the *k-*means++ algorithm to select the clustering center that can reflect the image features more so that the camouflage pattern design method in this paper can generate camouflage patterns with a better camouflage effect. Chapter 4 of this paper conducts the evaluation of the camouflage method. Chapter 5 of this paper conducts camouflage experiments, and this paper designs camouflage experiments under different weather conditions to verify the application value of the camouflage pattern generation method.

**Figure 1:**
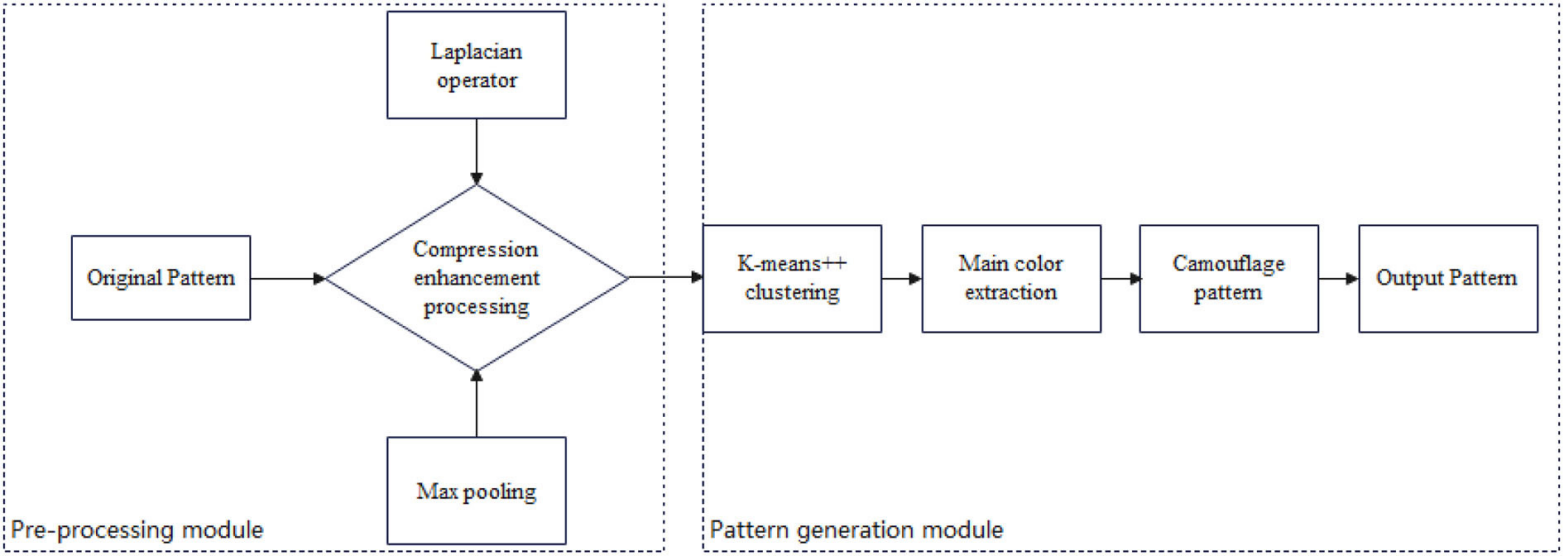
Overall design diagram.

The main contribution of this paper is to discover that the reason for the inaccurate results of the *k-*means traditional method for generating camouflage patterns is mainly that the *k-*means algorithm converges to a locally optimal solution when dealing with large and complex data, while camouflage patterns are mostly from aerial photographs of large and complex background patterns. The combination of the Max pooling-Laplacian algorithm and *k-*means++ is proposed to solve the problem that the camouflage pattern is not well-camouflaged enough. Figure 1 shows the overall design diagram.

## Image compression and enhancement based on Max pooling-Laplacian algorithm

In view of the different sources of background images, image quality and image content differ greatly, and the various types of background images acquired using high-definition lenses are rich in content and detail, which are detrimental to the *k-*means generated images and will affect the effectiveness of the program processing, it is necessary to compress and enhance the acquired data. Compression refers to reducing the image size while preserving the key features of the image, and enhancement is to perform differential operations on the data to sharpen the image and enhance the contrast. In this paper, the Max pooling-Laplacian algorithm is used for image compression and enhancement of the target background.

### Max pooling compression processing

**Figure 2:**
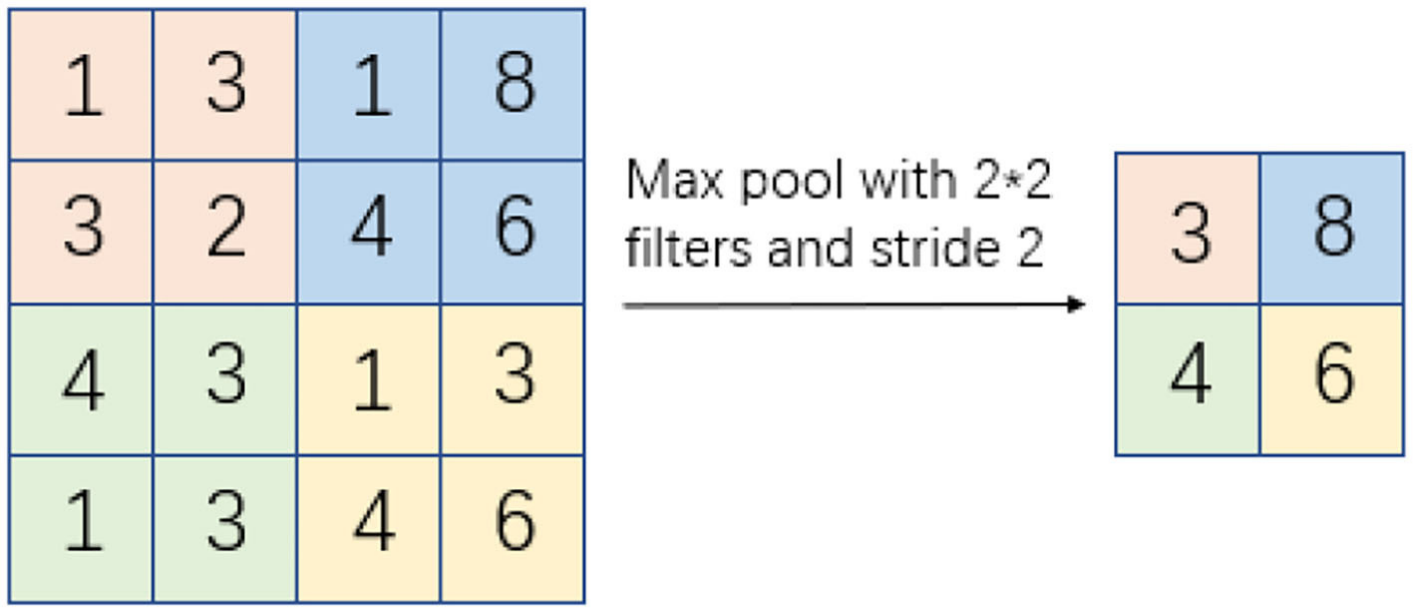
Pooling process.

Pooling refers to the pooling model (de Souza Brito et al., 2021), and pooling means that when computing the features of a region of an image, all the features of that region must be dissected and a new feature is used to represent all the features of the original region, which is also known as the neighborhood. Pooling has an obvious characteristic of reducing the size of the feature map while preserving the features, reducing the computational effort of the algorithm, and avoiding the phenomenon of computational overfitting. The pooling process is shown in Figure 2.

In Figure 2, the original feature image is a 4 x 4 matrix, from which it can be seen that the neighborhood is a 2 x 2 matrix with a pooling move step of 2, which eventually forms a new subsampled feature image of a 2 x 2 matrix. The pooling function *S*_0_ and the expressions are as follows:


(1) $$\begin{array}{l} \text{T }=\;{\text{S}}_{0}\left(\text{H}\right)+{\text{b}}_{2} \end{array}$$

where *T* is the subsampled feature map, *H* is the original feature map, and *b*_2_ is the resultant bias.

There are three common pooling operations: average pooling, Max pooling, and random pooling. Average pooling refers to summing the feature points in the neighborhood and taking the average as the subsampling feature value, and Max pooling refers to selecting the largest feature value in the pooling domain as the subsampling feature value. Random pooling refers to the random selection of features in the neighborhood according to the size of the probability value, and the larger feature value has a higher probability of being selected, unlike Max pooling where only the largest feature value is selected.

Compared with average pooling and random pooling, Max pooling can better preserve the texture features of the image and is more suitable for camouflage pattern design. In this paper, Max pooling is used to compress the image. The expression of Max pooling is where the moving step is set to *c* and(*H*_*ij*_) denotes the maximum feature value in the original feature map *H* of size *c* × *c* of the field captured the maximum feature value.


(2) $$\begin{array}{l} {\text{T}}_{\text{ij}}\;=\;\left({\text{H}}_{\text{ij}}\right)+{\text{b}}_{2} \end{array}$$

where *T*_*ij*_ is the subsampled feature map, *H*_*ij*_ is the original feature map, and *b*_2_ is the resultant bias.

### Laplacian image filtering enhancement processing

After the maximum pooling compression process, the efficiency of the program in processing the patterns is greatly improved, but the patterns after the compression process are reduced in clarity and content details. On the other hand, there is still noise in the four background patterns after the compression process that affects the processing results, which has a negative impact on the final processing effect. Noise removal is often done by filtering, which enhances the contrast and makes the processing effect more obvious. If the image is sharpened, it needs to be filtered, taking differential operations. In this paper, the filter template generated by the Laplace differential operator (Ilk et al., 2011) is chosen as the filter for sharpening the image background. The Laplace operator is defined as the scatter of the gradient of the function. Since the picture is a plane with only two directions x and y so set the function *f* (*x, y*).

The gradient of the function *f* (*x, y*) at the point (*x, y*) in the plane coordinate system:


(3) $$\begin{array}{l} \nabla \text{f}\left(\text{x},\text{y}\right)\;=\;\left\{\frac{\text{∂f}\left(\text{x},\text{y}\right)}{\text{∂x}},\frac{\text{∂f}\left(\text{x},\text{y}\right)}{\text{∂y}}\right\}=\;{\text{f}}_{\text{x}}\left(\text{x},\text{y}\right)\;•\;\overset{⇀}{\text{i}}+{\text{f}}_{\text{y}}\left(\text{x},\text{y}\right)\;•\;\overset{⇀}{\text{j}} \end{array}$$

The scatter of the function *f* (*x, y*) in the plane coordinate system:


(4) $$\begin{array}{l} \text{divf }=\;\nabla •\text{f }=\;\frac{\text{∂}{\text{f}}_{\text{x}}}{\text{∂x}}+\frac{\text{∂}{\text{f}}_{\text{y}}}{\text{∂y}} \end{array}$$

The function *f* (*x, y*) Laplace operator is defined as follows:


(5) $$\begin{array}{l} \nabla^{2}\text{f}\left(\text{x},\text{y}\right)\;=\;\frac{{\text{∂}}^{2}\text{f}\left(\text{x},\text{y}\right)}{\text{∂}{\text{x}}^{2}}+\frac{{\text{∂}}^{2}\text{f}\left(\text{x},\text{y}\right)}{\text{∂}{\text{y}}^{2}} \end{array}$$

where *x* and *y* represent the Cartesian coordinates of the *x*–*y* plane. In image processing, it is necessary to deform the general function Laplace operator to represent its discrete form in the *x, y* direction by the Laplace operator.

Discrete first-order differential equations:


(6) $$\begin{array}{l} \frac{\text{∂f}}{\text{∂x}}\;=\text{ f}\left(\text{x}+1\right)\;-\text{ f}\left(\text{x}\right) \end{array}$$

Discrete second-order differential equations:


(7) $$\begin{array}{l} \frac{{\text{∂}}^{2}\text{f}}{\text{∂}{\text{x}}^{2}}\;=\text{ f}\left(\text{x}+1\right)+\text{f}\left(\text{x}-1\right)-2\text{f}\left(\text{x}\right) \end{array}$$

The discretized Laplace operator is obtained and its approximate expression is shown in Equation 5.


(8) $$\begin{array}{l} \{\begin{array}{c} \frac{\partial^{2}\text{f}\left(\text{x},\text{y}\right)}{\partial {\text{x}}^{2}}=\text{f}\left(\text{x}+\text{1},\text{y}\right)+\text{f}\left(\text{x}-\text{1},\text{y}\right)-2\text{f}\left(\text{x},\text{y}\right)\\ \frac{\partial^{2}\text{f}\left(\text{x},\text{y}\right)}{\partial {\text{y}}^{2}}=\text{f}\left(\text{x},\text{y}+\text{1}\right)+\text{f}\left(\text{x},\text{y}-\text{1}\right)-2\text{f}\left(\text{x},\text{y}\right) \end{array} \end{array}$$

Substituting Equation 8 into Equation 5 yields Equation 9:


(9) $$\begin{array}{l} \nabla^{2}\text{f}\left(\text{x},\text{y}\right)=\text{f}\left(\text{x}+1,\text{y}\right)+\text{f}\left(\text{x}-1,\text{y}\right)+\text{f}\left(\text{x},\text{y}+1\right)\\ +\text{f}\left(\text{x},\text{y}-1\right)-4\text{f}\left(\text{x},\text{y}\right) \end{array}$$

**Figure 3:**
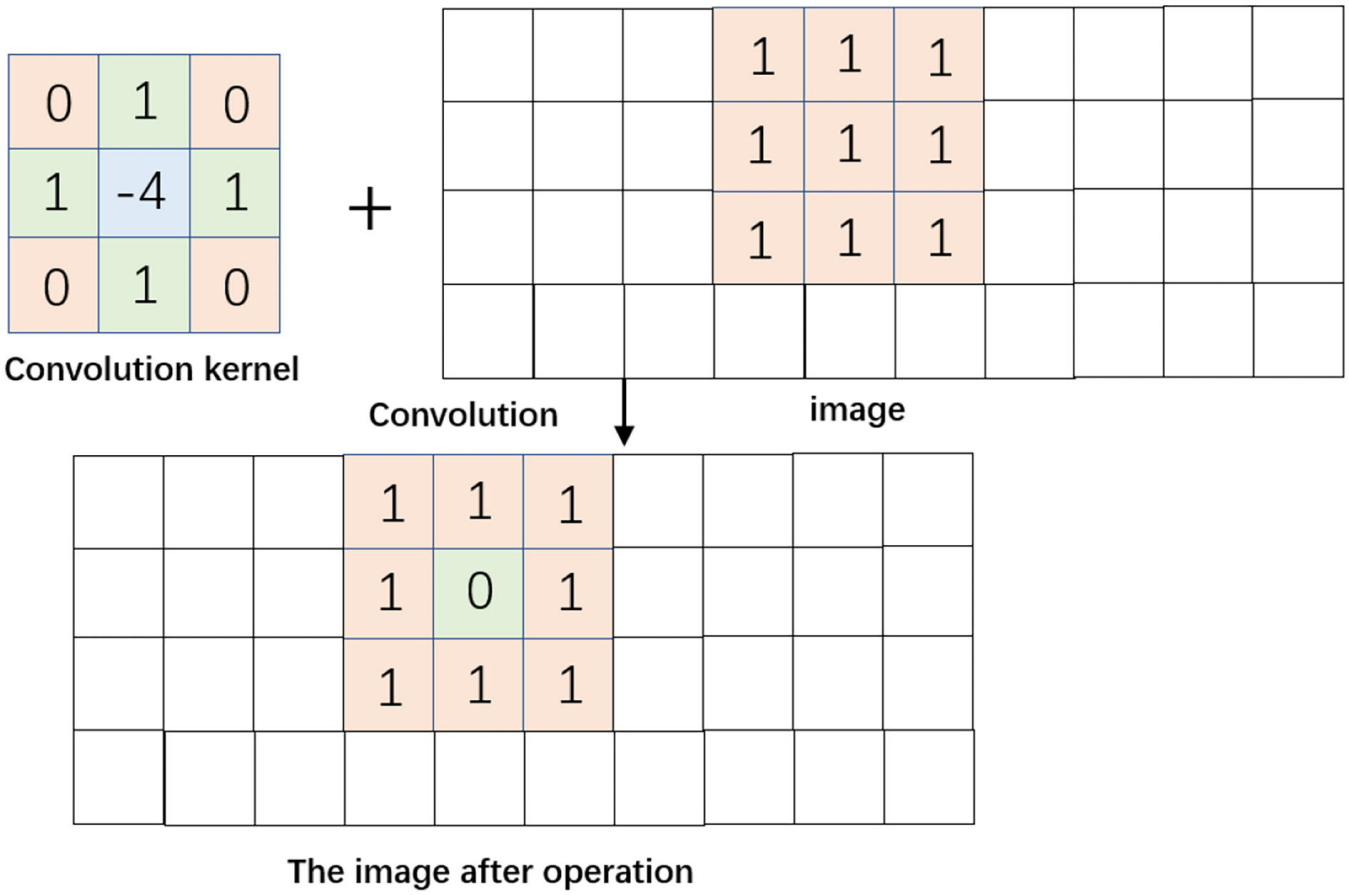
Template and image convolution process.

The above equation represents the discrete form of the Laplace operator in both the x and y directions. A filtering template evolved from the discretized Laplace differential operator is slid over each point in the space to realize the convolution operation of the image and the template to achieve the purpose of image filtering and enhancement. The implementation process is as follows:

Figure 3 shows the process of convolution of the template with the image. The template is overlapped with the image, a region of pixels equal to its size and the corresponding pixel points are found, then the operation is performed with the region, the values in the template are summed with the values of the corresponding pixel points, and the whole is summed again, and the result is assigned to the pixel overlapped with the center point of the template. After that, the template is slid to the next position and the above operation is repeated until all pixels are traversed. In Figure 3, the resulting centroid pixel value is converted from “1” to “0”. If there are negative values in the Laplace filter template, the negative values will be intercepted and set to 0 after filtering, which will result in the loss of some grayscale information in the image data filtering. In order to solve this problem, the lost grayscale information can be restored by subtracting the filtered data from the original background, and thus the basic formula of the Laplace operator image enhancement is shown in Equation 10:


(10) $$\begin{array}{l} \text{G}\left(\text{x},\text{y}\right)\;=\text{ f}\left(\text{x},\text{y}\right)+\text{c}\left[\nabla 2\text{f}\left(\text{x},\text{y}\right)\right] \end{array}$$

In the above equation, *g*(*x,y*) is the enhanced background patterns, *f* (*x,y*) is the original background patterns, and *c* is related to the template center coefficient; if positive, *c* is 1, and if negative, *c* is -1.

## Camouflage generation based on the *k-*means clustering algorithm

### *k*-means clustering algorithm

**Figure 4:**
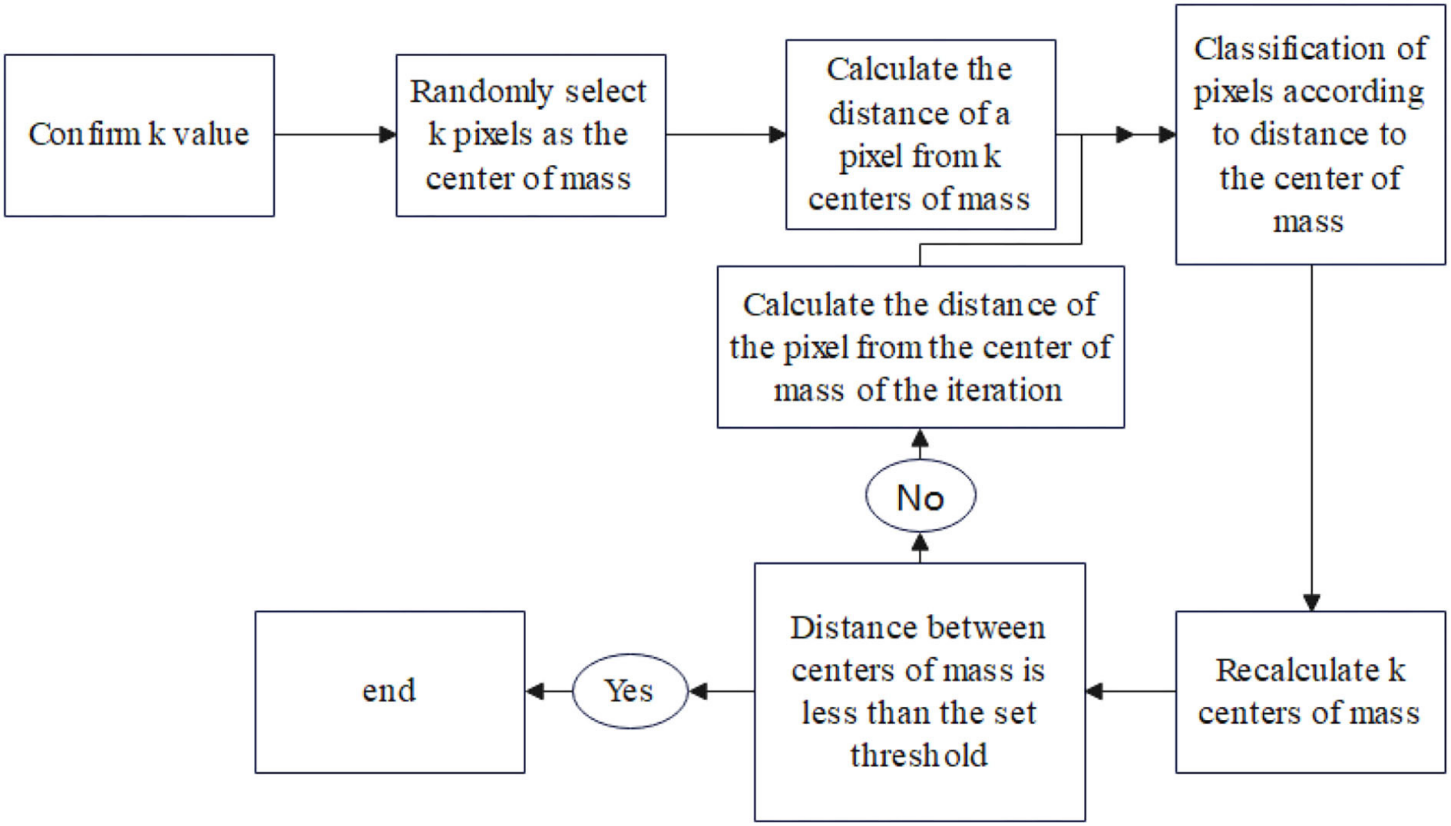
*k-*means algorithm clustering block diagram.

The *k-*means algorithm (Kövesi et al., 2001) is one of the most widely used algorithms in cluster analysis. The *k* in the *k-*means algorithm means that the clusters are *k* clusters, and the mean of the data values in each cluster is taken as the center of the cluster, or the center of mass, i.e., the center of mass of each class is used to describe the cluster. The *k-*means algorithm steps are shown in Table 1. Figure 4 shows the clustering block diagram of the *k-*means algorithm.

**Table 1:** *k-*means algorithm.

***k*** **-means algorithm steps**	
Step 1	Randomly select *K* samples from the dataset as the initial cluster center *C* = {*c*_1_, *c*_2_, …, *c*_*k*_}
Step 2	For each sample x in the dataset, calculate its distance to *K* cluster centers and divide it into clusters corresponding to the cluster center with the smallest distance
Step 3	For each cluster, recalculate its cluster center $c_{i}=\frac{1}{\left|c_{i}\right|}\underset{x\in c_{i}}{\sum }x$
Step 4	Repeat Step 2 and Step 3 until the cluster center does not change

The advantages of the *k-*means clustering algorithm are as follows:

1. The principle of the algorithm is simple, and it is easy to apply.
2. The convergence speed is faster and the clustering effect is better when dealing with regular data.

The disadvantages of the *k-*means algorithm are as follows:

1. The selection of the initial clustering centroids is random, and it may select bad initial values, which may have bad effects on the clustering speed and results afterward.
2. When dealing with complex data, the iterative results are not accurate only the local optimal solution, it is difficult to obtain the global optimal solution.
3. Very easy to be affected by noise and outliers.

### *k*-means++ clustering algorithm

The biggest difference between *k-*means++ (Arthur and Vassilvitskii, 2006) and the *k-*means algorithm is in the selection of cluster centers. The *k-*means algorithm selects cluster centers randomly, while the *k-*means++ algorithm selects cluster centers with the core idea that the initial cluster centers should be as far away from each other as possible, which is more in line with the human intuition to select cluster centers. Table 2 shows the steps of the *k-*means++ algorithm.

**Table 2:** *k-*means++ algorithm.

***k*****-means**++ **algorithm steps**	
Step 1	Randomly select a sample from the dataset as the initial clustering center *c*_1_
Step 2	First calculate the shortest distance *D*_(*i*)_ between each sample and the currently existing clustering center and then calculate the probability *p*_*i*_($p_{i}=\frac{{D_{\left(i\right)}}^{2}}{\underset{i\in x}{\sum }{D_{\left(i\right)}}^{2}}$) that each sample is selected as the next clustering center. Finally, the next clustering center is selected based on the roulette wheel method.
Step 3	Repeat Step 2 until all cluster centers are selected
The subsequent steps are the same as Steps 2–4 of the *k-*means algorithm	

Although the *k-*means++ algorithm takes time to select the clustering centers, it converges faster in subsequent iterations and the results are more accurate. In general, it takes less time to compute and has less error than the *k-*means algorithm.

### The camouflage generation process of the *k-*means clustering algorithm

**Figure 5:**
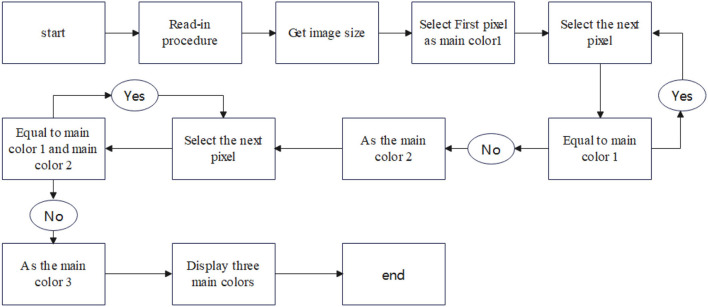
Block diagram of main color extraction program.

The key steps are shown below:

1. Construct the clustering machine. Set the initial *k* clustering centers, i.e., the generated clusters are of type *k*; the maximum number of iterations to perform one *k-*means algorithm is set to 4,000; the initialization specifies the method with three alternative values.
2. The default value of “*k-*mean++”, “random”, and passing array vectors is chosen to speed up the iterative convergence process, reduce the loss and improve the efficiency. Among the above settings, the most influential one on the final results is the number of cluster centers *k*, which directly determines the type of camouflage colors generated and will be further discussed in the subsequent work.
3. Obtain the clustering centers for primary color extraction. After the background clustering is finished, the clustering results are further processed to obtain relevant clustering information, including the values of each clustering center and other attributes. The specific extraction procedure is shown in Figure 5.
4. The program runs in blocks. In this paper, four types of backgrounds are placed in one project during the design process, and different background types perform different operations, thus different program codes need to be selected for different background types during the running process in order to get the correct results.
5. Result optimization and refinement. Optimize the overall camouflage effect of the camouflage pattern by adjusting the number of clustering centers to find the best *k-*value.

In order to verify the significance of the algorithm in practical applications, specific examples are designed in this paper for validation. In this paper, four backgrounds such as woodland background, grassland background, desert background, and snowy background are selected as the test images.

**Figure 6:**
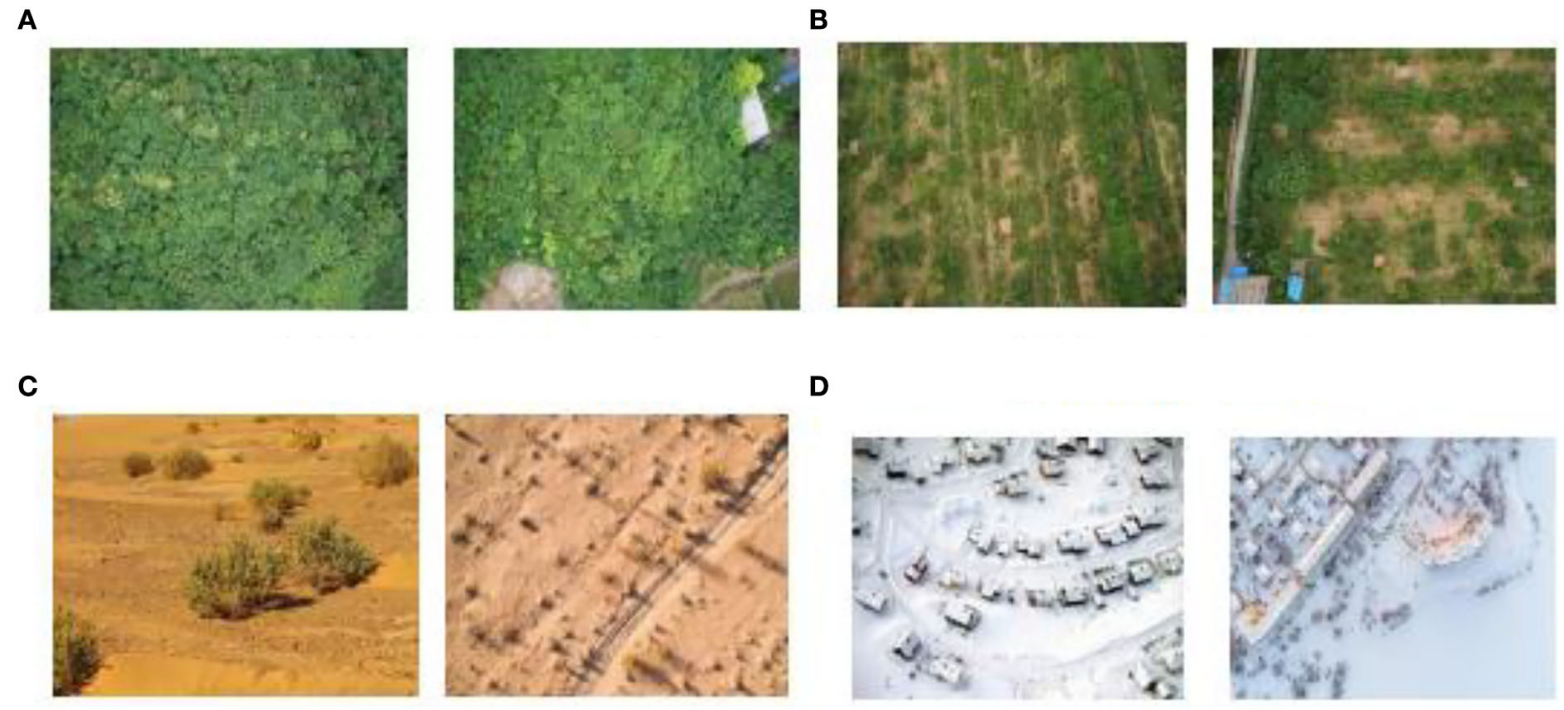
Background data image. **(A)** Woodland background, **(B)** grass background, **(C)** desert background, **(D)** snowy background.

Figure 6 shows the selected backgrounds. These images are processed in two different ways to generate the final camouflage pattern with the Max pooling-Laplacian algorithm pre-processed clustering and the camouflage pattern without pre-processed clustering. The strengths and weaknesses of the algorithm-generated results are compared by evaluating the similarity of these two camouflage patterns to the background of the original image. Pictures **(A)** and **(B)** in Figure 6 are taken by individuals and Pictures **(C)** and **(D)** were purchased and downloaded from the goadingsucai website.

## Results and Discussion

### Design example

**Figure 7:**
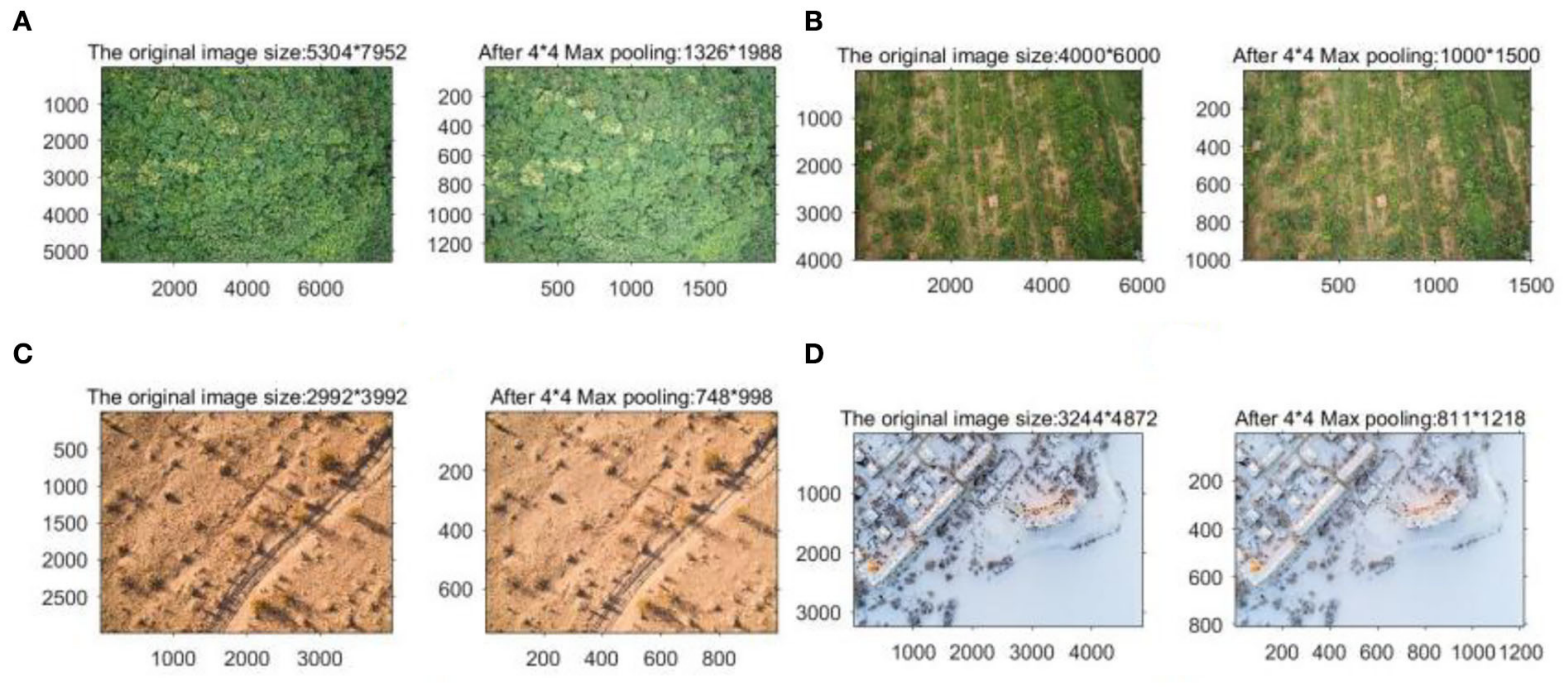
Before and after pooling comparison: **(A)** woodland background; **(B)** grass background; **(C)** snow background; and **(D)** desert background.

The results of the background image obtained by processing using the Max pooling model with a step size of 4 and a domain of 4 x 4 are shown in Figure 7.

**Figure 8:**
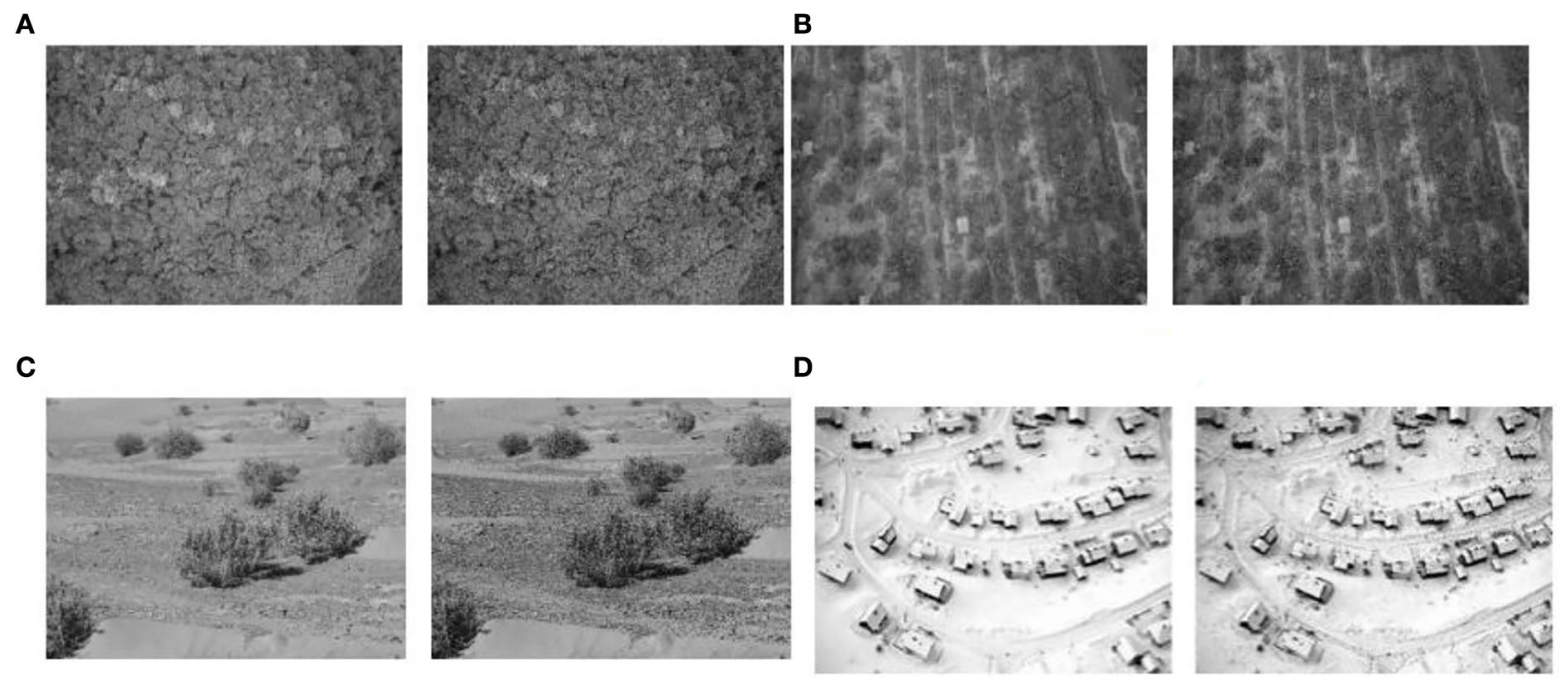
Comparison of before and after background processing: **(A)** woodland background; **(B)** grass background; **(C)** desert background; and **(D)** snowy background.

As can be seen from Figure 7, there is a certain decrease in the clarity of the background image after pooling, but it can still retain the texture features of the original image better; at the same time, the size of the image decreases significantly, which can reduce the computation of the program, and the image is enhanced by Laplace operator filtering as shown in Figure 8.

In Figure 8, the grayscale background is shown on the left, and the grayscale background processed by Laplace arithmetic filtering is shown on the right. The comparison between the grayscale background and the filtered background shows that the filtering process increases the contrast between the grayscale background, the background texture contour becomes clearer, and the sharpening effect is achieved.

The camouflage pattern generated from the unprocessed background image and the camouflage pattern with degraded quality and noise reduction is selected for comparison.

**Figure 9:**
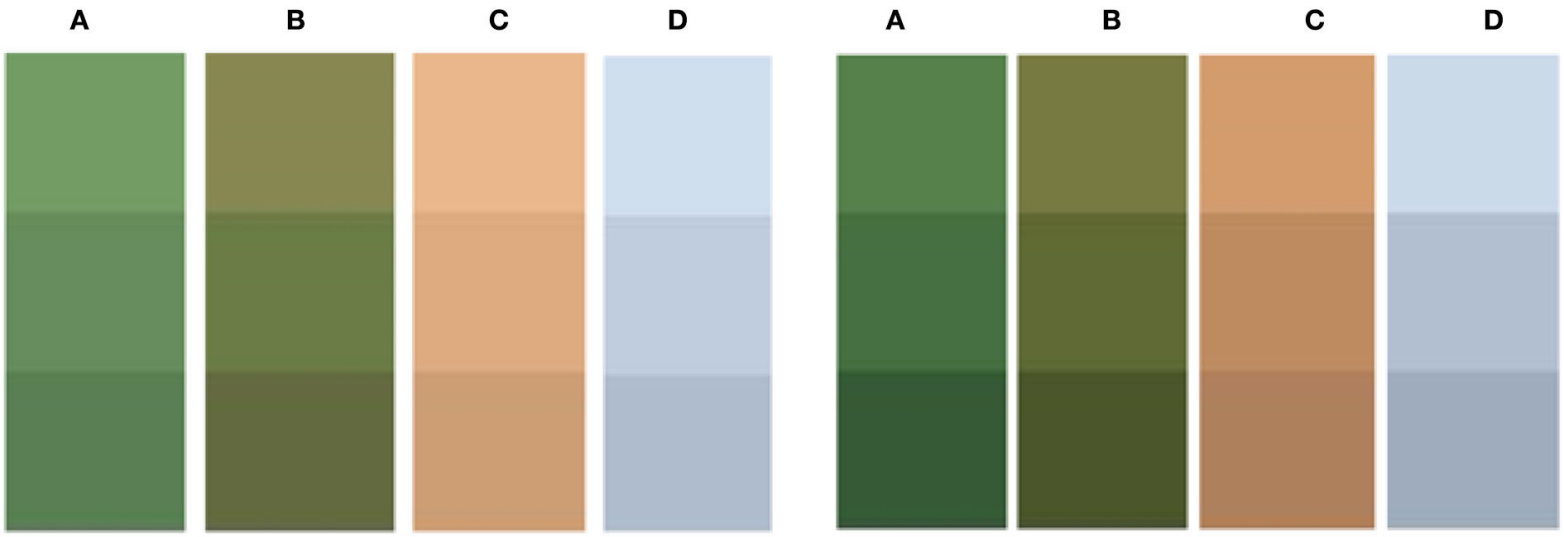
Max pooling-Laplacian pre-processed primary color and un-pre-processed primary color **(A)** woodland background; **(B)** grassland background; **(C)** desert background; and **(D)** snow background.

**Figure 10:**
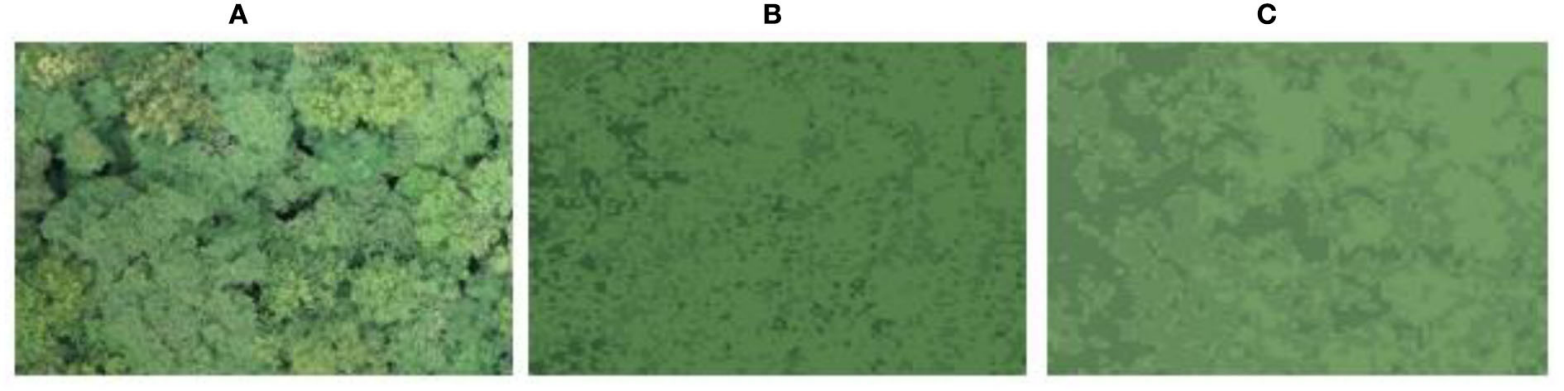
Woodland-type camouflage pattern. **(A)** Original image, **(B)** unprepared, **(C)** compressed and enhanced.

According to the process and procedure of primary color extraction, the corresponding procedure was written in the software platform, and the value of *k* was set to 3. The primary color maps of the camouflage design patterns corresponding to the camouflage without image processing and after processing by the Max pooling-Laplacian algorithm and the RGB values of each color were obtained, respectively, and the summary results are shown in Figures 9, 10 and Table 3.

**Table 3:** Main color extraction table.

**Background type**	**Not pre-processed**			**Max pooling-laplacian preprocessing**		
	**Main color 1**	**Main color 2**	**Main color 3**	**Main color 1**	**Main color 2**	**Main color 3**
**Woodland**	86,129,75	70,112,66	54,90,54	115,156,100	103,141,94	90,127,84
**Grassland**	119,122,65	96,106,53	75,86,43	135,135,81	107,124,70	99,106,64
**Desert**	210,156,109	190,139,96	174,128,92	234,183,140	222,171,128	205,158,116
**Snow**	205,158,116	178,191,208	159,172,189	207,223,238	192,205,222	175,188,205

**Figure 11:**
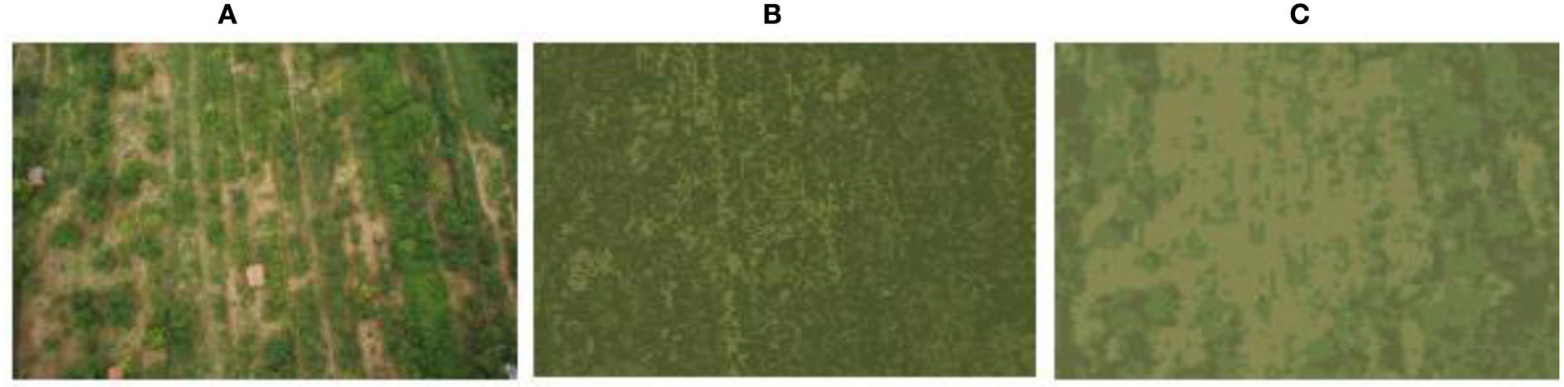
Grass-type camouflage pattern. **(A)** Original image, **(B)** unprepared, **(C)** compressed and enhanced.

**Figure 12:**
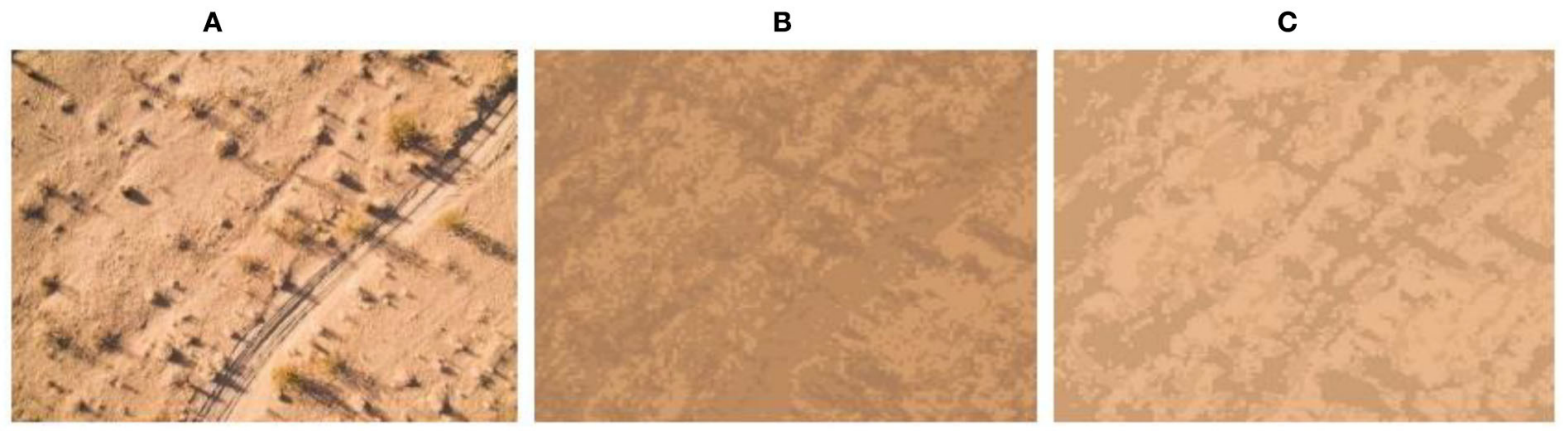
Desert-type camouflage pattern. **(A)** Original image, **(B)** unprepared, **(C)** compressed and enhanced.

**Figure 13:**
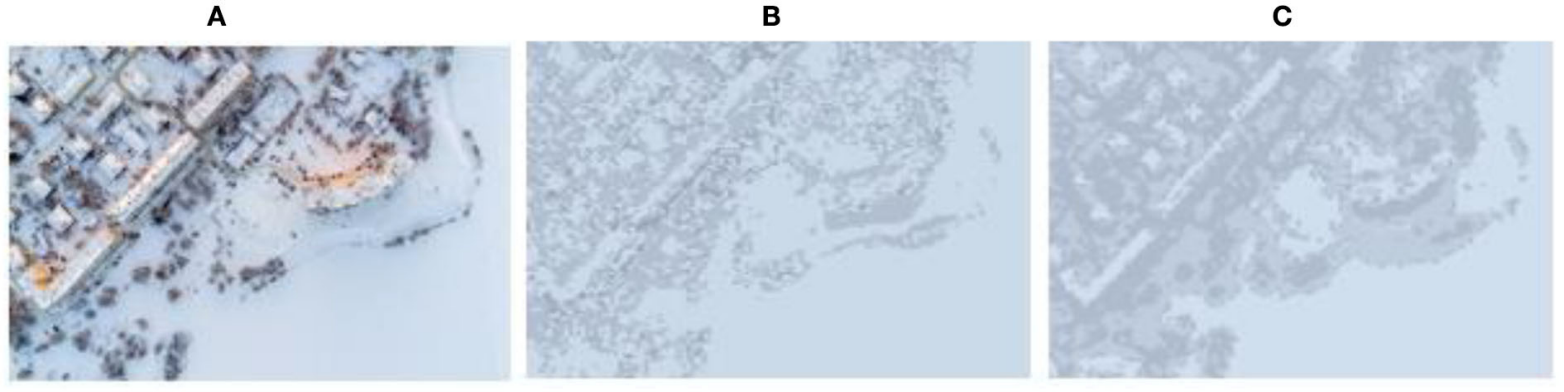
Snow-type camouflage pattern. **(A)** Original image, **(B)** unprepared, **(C)** compressed and enhanced.

Figures 10–13 show the results of camouflage pattern production for woodland, grass, desert, and snow backgrounds, respectively. Since the camouflage pattern outputted by setting the *k-*value to 3 is tricolor camouflage, which are the primary, secondary, and transitional colors of the image, respectively, tricolor camouflage is more widely used in camouflage in various countries. From the image results, the camouflage pattern generated by the background image after Max pooling-Laplacian pre-processing is closer to the overall texture and overall color distribution of the original background image than the unprocessed camouflage pattern, especially in the background mottled feature-rich woodland background pattern and grass background pattern, and the effect of the camouflage pattern generated by the pre-processing is obviously better.

### Camouflage effect evaluation

This paper introduces cosine similarity cosθ theory (Xia et al., 2015) and Euclidean distance *d*_1 − 2_ (Li and Lu, 2009) to evaluate the effect of camouflage pattern generation.

The cosine similarity is judged by the cosine value of the spatial vector angle, and the Euclidean distance *d*_1 − 2_ reflects the degree of similarity by the size of the mode of the difference between two vectors, where the cosine similarity is closer to 1 and the smaller Euclidean distance *d*_1 − 2_ means the camouflage generated pattern is closer to the background pattern.

If the vector α $=\left[\begin{array}{c} x_{1}\&y_{1}\&z_{1} \end{array}\right]$, $b=\left[\begin{array}{c} x_{2}\&y_{2}\&z_{2} \end{array}\right]$, α*b* is the cosine of the angle between cosθ and the Euclidean distance *d*_1 − 2_ are as follows:


(11) $$\begin{array}{l} \cos\text{θ}=\frac{{\text{x}}_{1}{\text{x}}_{2}+{\text{y}}_{1}{\text{y}}_{2}+{\text{z}}_{1}{\text{z}}_{2}}{\sqrt{{\text{x}}_{1}^{2}+{\text{y}}_{1}^{2}+{\text{z}}_{1}^{2}}\sqrt{{\text{x}}_{2}^{2}+{\text{y}}_{2}^{2}+{\text{z}}_{2}^{2}}} \end{array}$$

(12) $$\begin{array}{l} {\text{d}}_{1-2}=\sqrt{{\left({\text{x}}_{1}-{\text{x}}_{2}\right)}^{2}+{\left({\text{y}}_{1}-{\text{y}}_{2}\right)}^{2}+{\left({\text{z}}_{1}-{\text{z}}_{2}\right)}^{2}} \end{array}$$

where α* and b* are the two spatial vectors and θ is the angle between the two vectors.

In terms of color similarity, HSV color space is the most recent human subjective recognition color space, which consists of three components: *H* (hue), *S* (saturation), and *V* (Value) (Cai et al., 2012). Since the picture information in HSV color space is better than RGB color space, the HSV color space vector is chosen to be *h*, *s*, *v* components are involved in the cosine distance; and Euclidean distance calculation. The range of values of *H* is 0–360, the range of values of *S* is 0–100, and the range of values of *V* is 0–100.

In shape similarity image shape features are often described by image shape invariant moments and moment sets, which can describe both the global features of an image and provide information about different geometric features related to that image, including the characteristics of size, direction, and shape (Dai et al., 2021). The invariant moments φ1, φ2, and φ3 derived from them are selected as evaluation metrics to construct the invariant moment set feature vector and calculate the Euclidean distance and cosine distance between the two.

The final output color similarities and shape similarities data are summarized in Tables 4, 5. From the data table, we can see that the *k-*means clustered camouflage pattern output after Max pooling-Laplacian compression enhancement process has a cosine similarity closer to 1 than the unprocessed camouflage pattern output. *The*cosθ is closer to 1 and the Euclidean distance *d*_1 − 2_ is smaller.

**Table 4:** The color similarity of camouflage patterns.

**Background type**	**Pattern type**	**H**	**S**	**V**	**cosθ**	***d*_1 − 2_**
**Woodland**	Not pre-processed	100.65	40.63	62.73	0.9957	11.68
	Original image	108.12	36.36	54.85		
					0.9999	2.685
	Compression enhancement processing	109.73	37.88	56.37		
**Grassland**	Not pre-processed	70.019	42.28	48.8	0.9930	11.41
	Original image	67.72	52.57	44.42		
					0.9998	3.2
	Compression enhancement processing	66.42	49.65	44.18		
**Desert**	Not pre-processed	30.9	40.68	85.74	0.9986	5.78
	Original image	27.10	43.41	82.34		
					0.9996	3.35
	Compression enhancement processing	27.89	46.60	83.01		
**Snow**	Not pre-processed	216.69	19.4	78.45	0.9996	7.82
	Original image	210.37	15.38	80.69		
					0.9999	3.22
	Compression enhancement processing	213.20	16.8	81.23		

**Table 5:** Shape similarity of camouflage patterns.

**Background type**	**Pattern type**	**φ1**	**φ2**	**φ3**	**cosθ**	***d*_1 − 2_**
**Woodland**	Not pre-processed	0.2208	0.0097	0.0004	0.9996	0.06
	Original image	0.2775	0.0082	0.0002		
					0.9999	0.02
	Compression enhancement processing	0.2545	0.0093	0.0002		
**Grassland**	Not pre-processed	0.2633	0.0034	0.0001	0.9998	0.05
	Original image	0.3122	0.0083	0.0010		
					0.9999	0.01
	Compression enhancement processing	0.3003	0.0080	0.0014		
**Desert**	Not pre-processed	0.5377	0.0266	0.006	0.9994	0.06
	Original image	0.2201	0.004	0		
					0.9998	0.02
	Compression enhancement processing	0.2409	0.0042	0.0001		
**Snow**	Not pre-processed	0.2808	0.0097	0.0004	0.9998	0.06
	Original image	0.2208	0.0072	0.0001		
					0.9999	0.02
	Compression enhancement processing	0.2421	0.0083	0.0002		

**Figure 14:**
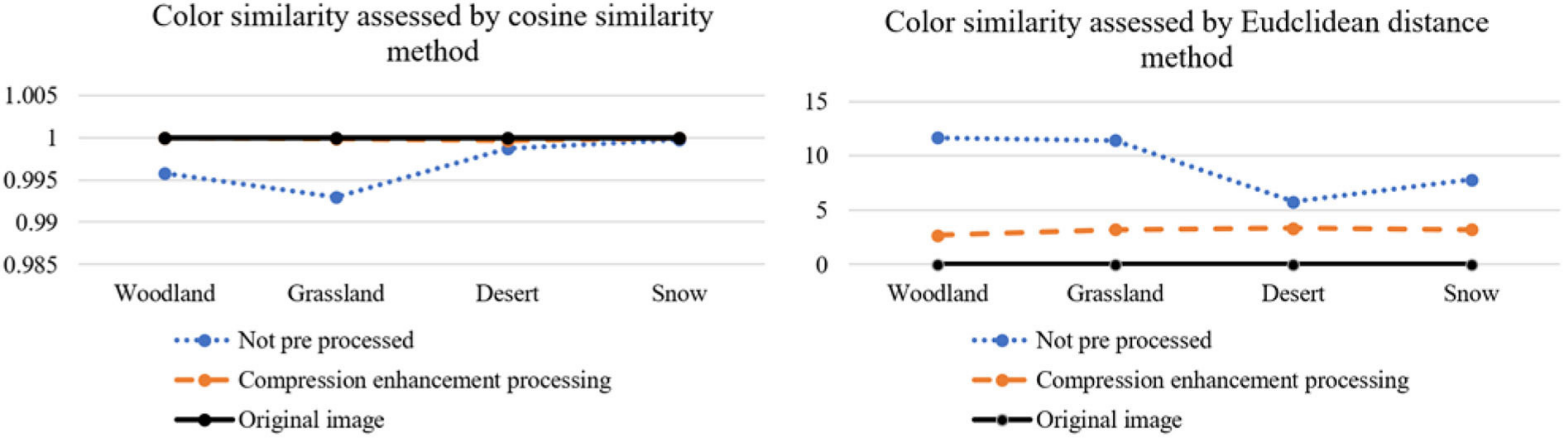
Color similarity assessment results.

**Figure 15:**
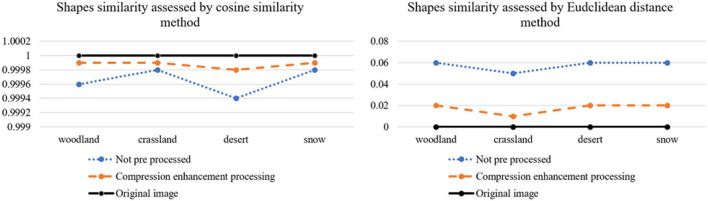
Shapes similarity assessment results.

From Figures 14, 15, it can be visually analyzed that the camouflage pattern using the maximum pooling-Laplace compression enhancement algorithm is closer to the original image. It can be concluded that the design method of using the Max pooling-Laplacian algorithm proposed in this paper to pre-process images and then *k-*means clustering to generate camouflage patterns can be better.

## Practical application verification

### Experimental design

In order to verify the application value of camouflage design theory and view the camouflage effect of camouflage patterns in the actual environment. In this paper, the camouflage effect of the camouflage design pattern is verified by combining the existing conditions and printing the camouflage design pattern with a grassland background on the common fabric. The grass background camouflage pattern generated by the algorithm of this paper is selected as the camouflage pattern of the test piece. The three main colors extracted from the grassland background at *k* = 3 were selected as the main colors of the test piece.

**Figure 16:**
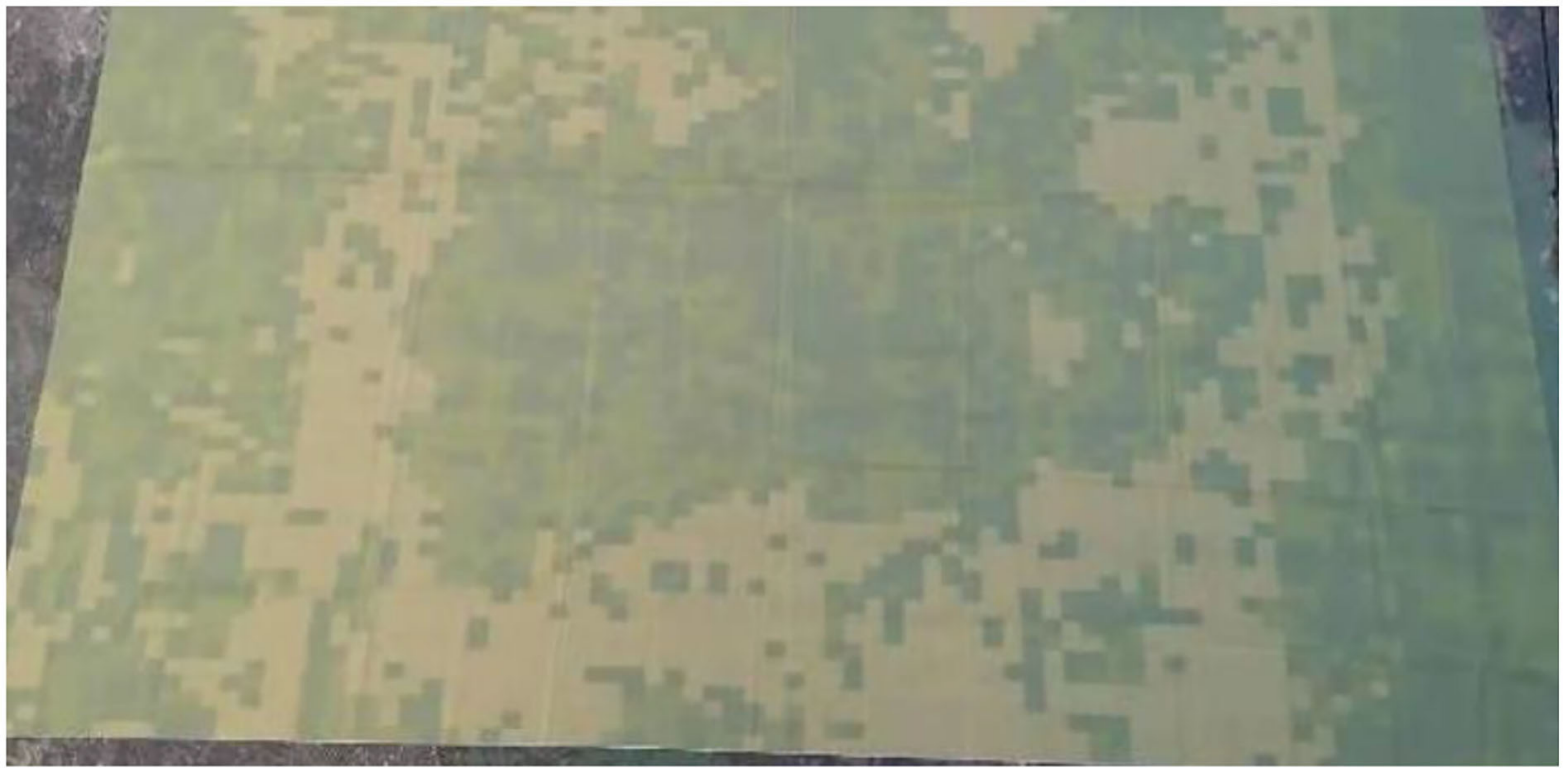
1 * 0.6 m test piece.

The camouflage pattern is printed on the fabric carrier to realize the camouflage design materialization, and the size of the test piece is 1 ^*^ 0.6 m, as shown in Figure 16.

**Figure 17:**
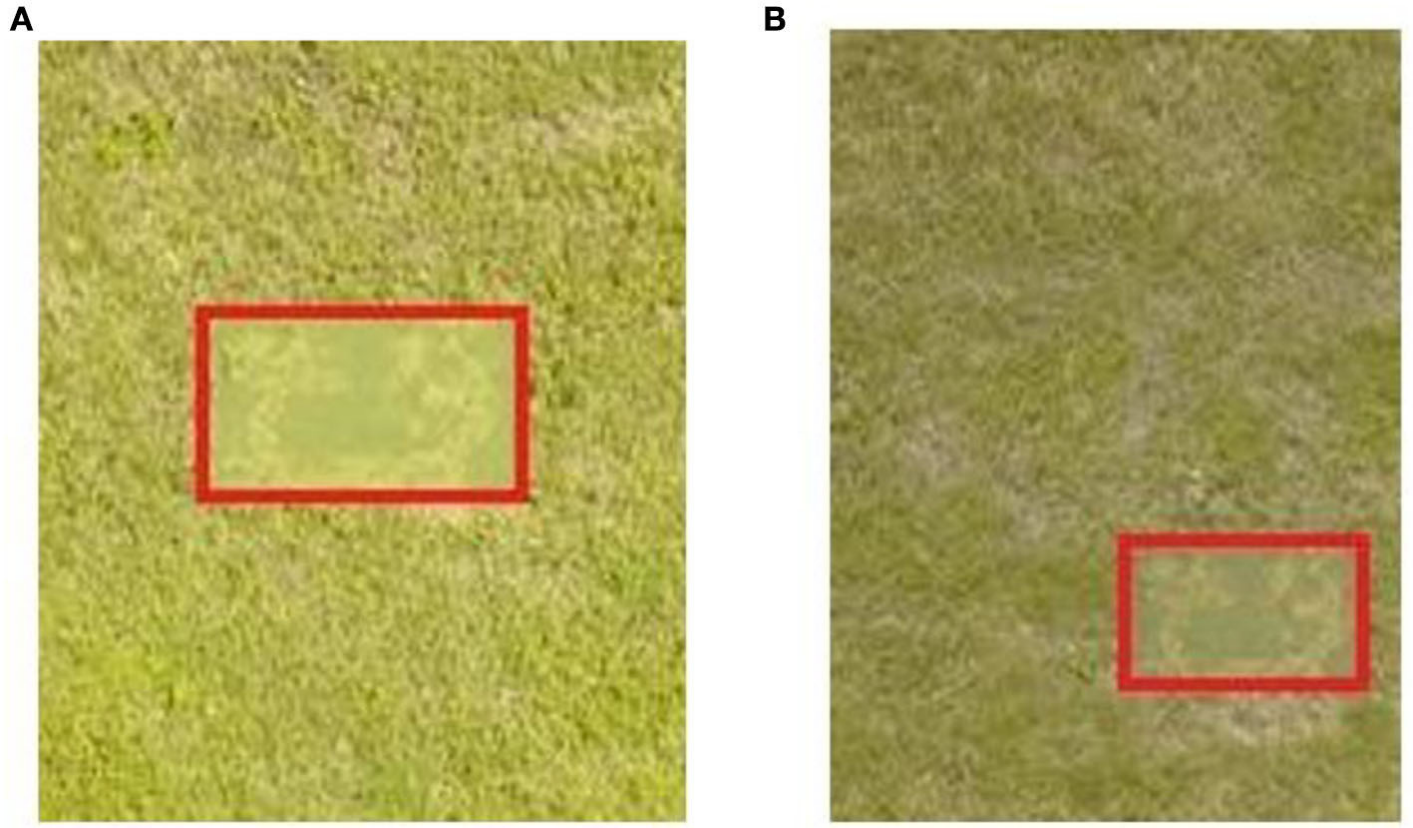
Test pieces under different meteorological conditions. **(A)** Sunny, **(B)** cloudy.

In this paper, we set the shooting height to 25 m and use DJI Genie 4pro to shoot. In the actual testing process, factors such as moisture in the air, visibility, and weather conditions will have a great impact on the testing effect and bring influence to the evaluation of the designed camouflage pattern. In this paper, the camouflage effect was tested under two different weather conditions, sunny and cloudy, and the results are shown in Figure 17.

As can be seen from Figure 17, the results of the test piece camouflage effect detection under two different meteorological conditions show obvious differences. Whether it is the color of the image or the brightness of the image, the results obtained under different meteorological conditions are obviously different results.

### Analysis of experimental results

**Figure 18:**
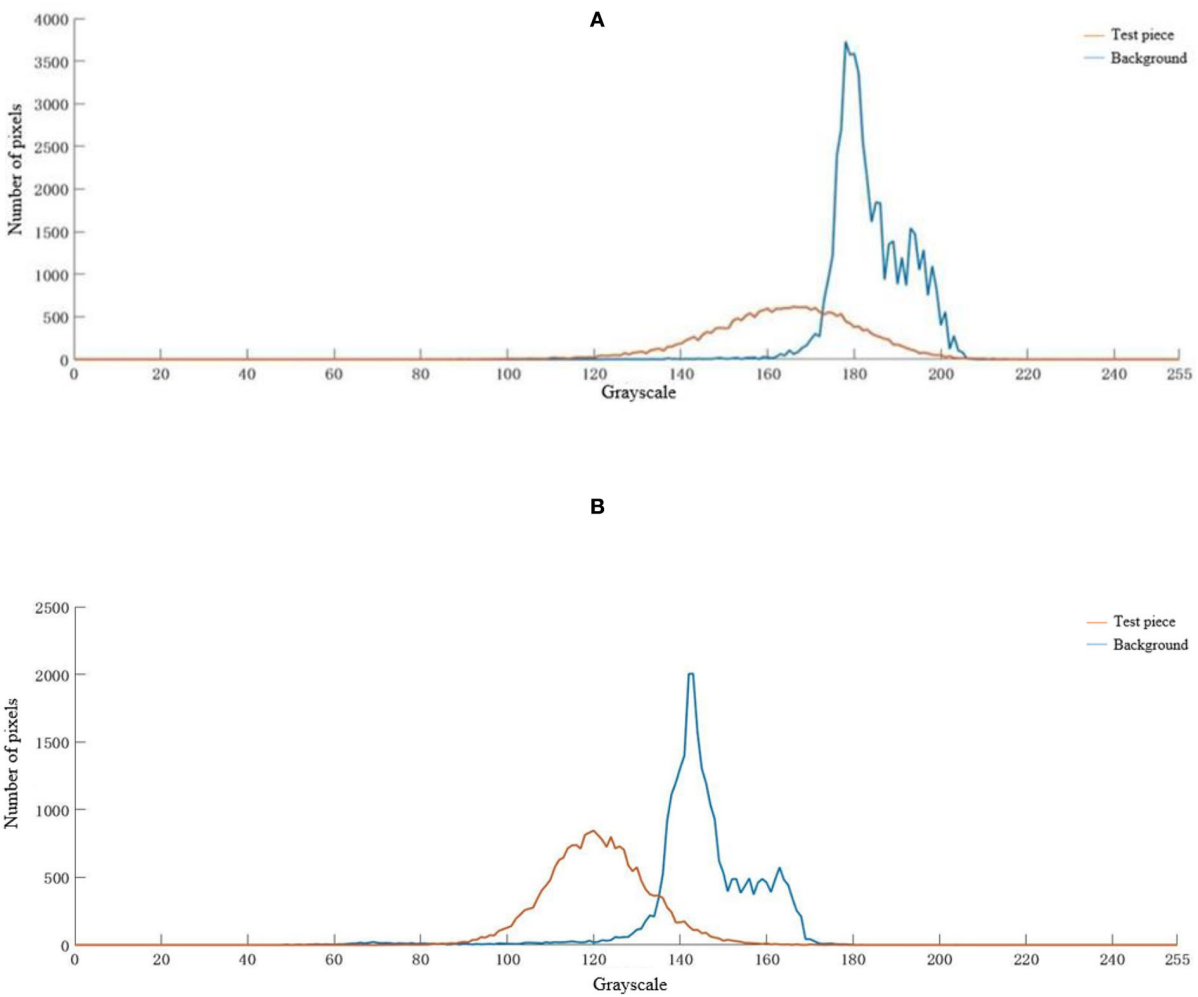
Color histogram of test piece and background under different meteorological conditions. **(A)** Sunny, **(B)** cloudy.

In Figure 18, the color histogram distribution between the test piece and its background under two different weather conditions, sunny and cloudy, is somewhat different. Under sunny weather conditions, the gray level of the test piece is mainly distributed in the interval [140, 180] and peaks at the gray level of 170, and the gray level of the background is mainly distributed in the interval [170,190] and peaks at the gray level of 180. Under cloudy weather conditions, the test piece gray levels are mainly distributed in the interval [110, 140], and the gray level peaks at 120; the background gray levels are mainly distributed in the interval [130, 150], and the gray level peaks at 140. The test pieces and the actual background gray level intervals overlap well, and the differences in the gray levels corresponding to the peaks are small. This experimental result proves that the camouflage pattern generated by the camouflage pattern generation method in this paper has a good camouflage effect.

Comparing the distribution of the histogram of the test piece with its background color under two different meteorological conditions of sunny and cloudy days. The gray level region slides from the high gray level in sunny weather conditions to the low gray level in cloudy weather conditions, which indicates that the weather conditions have some influence on the camouflage effect of camouflage. The gray level is closely related to the image brightness, and sufficient light will increase the brightness of the test piece and the background, which will bring some influence to the camouflage effect.

From Table 6, it can be seen that the color difference between the test piece and the background image is minimal for either meteorological condition, which indicates that the method in this paper achieves a better effect in color reproduction. The differences in Euclidean distance and cosine similarity show that the meteorological conditions can have an effect on the artifact effect. The Euclidean distance under sunny weather conditions is greater than that under cloudy weather conditions, indicating that sufficient light causes the test piece and the background to change in the direction of brightness enhancement, which weakens the camouflage effect of the test piece. This is because the brightness is closely related to the spectral reflectance characteristics of the material, and the common fabric selected in this paper is not specially treated, resulting in significant differences in the brightness of the target background, making the camouflage effect weaker. Therefore, the material properties of the coating or fabric need to be taken into consideration when camouflage is implemented.

**Table 6:** The similarity between the test piece and background under different meteorological conditions.

**Weather conditions**	**Image type**	***h***	***s***	***v***	***d*_1 − 2_**	***cosθ***
**Sunny**	Background	59.06	57.59	70.43	11.92	0.9941
	Test piece	60.83	47.98	77.26		
**Cloudy**	Background	53.53	46.54	55.70	7.54	0.9965
	Test piece	55.28	40.41	60.04		

The above experimental analysis shows that the test pieces have good camouflage effects under different weather conditions. The high degree of integration between the test piece and the background, good similarity, and good camouflage effect further illustrate the application value of the camouflage pattern generation algorithm designed in this paper. It also indirectly proves that good lighting conditions will enhance the contrast between the ground target and background and weaken the comprehensive camouflage effect of camouflage.

## Conclusion

In this paper, the Max pooling-Laplacian algorithm is used to process the original background images based on different background data to achieve the design of camouflage patterns with different background data. Compared with the traditional *k-*means clustering camouflage pattern method, the method in this paper has a better camouflage effect. Experiments were designed to verify the application value of the camouflage design method, and the final experimental results showed that the camouflage design method has good reliability.

(1) In this paper, the Max pooling theory is applied to compress the images to reduce the image size while preserving the image features, speed up the camouflage pattern generation, and prevent too much information from causing inaccurate convergence results.
(2) A Laplace filter was used to enhance the image operation, which weakened the influence of image clutter on the design results and improved the accuracy of the algorithm results.
(3) The design method is evaluated by using color similarity and shape similarity, and the results show that the new camouflage pattern generation method achieves the design of camouflage patterns with different backgrounds and achieves good results.
(4) Camouflage experiments under different weather conditions were designed. Under sunny conditions, the cosine similarity between the camouflage pattern and background was 0.9941 and the Euclidean distance was 11.92; under cloudy conditions, the cosine similarity between the camouflage pattern and background was 0.9965 and the Euclidean distance was 7.52.

Although the camouflage design method in this paper is better than the traditional *k-*means camouflage design method. However, the data from Figures 14, 15 reflect that the camouflage effect of the woodland background camouflage picture and the grass background camouflage pattern generated by the design method in this paper is better. This is because the data of snow background and desert background are not as complex as the data of woodland background and grass background, and the method designed in this paper is more suitable for complex data images and large data images. The results of the camouflage experiments in this paper show that different lighting conditions can affect the brightness of the background and camouflage patterns resulting in a poor camouflage effect. Future research can focus on camouflage pattern materials and coatings, and design materials with a similar reflectance to the background spectrum.

## Data availability statement

The original contributions presented in the study are included in the article/supplementary material, further inquiries can be directed to the corresponding author/s.

## Author contributions

MW and DZ provided research ideas and plans. MW improved the algorithm. MW and BZ wrote the programs and conducted the experiments. BZ was responsible for collecting data. MW wrote the manuscript with the help of DZ. DZ revised the manuscript and approved the final submission. All authors contributed to the article and approved the submitted version.

## Conflict of interest

The authors declare that the research was conducted in the absence of any commercial or financial relationships that could be construed as a potential conflict of interest.

## Publisher's note

All claims expressed in this article are solely those of the authors and do not necessarily represent those of their affiliated organizations, or those of the publisher, the editors and the reviewers. Any product that may be evaluated in this article, or claim that may be made by its manufacturer, is not guaranteed or endorsed by the publisher.
